# Investigating Differences in Patients Enrolled in a Clinical Study Based on Referral Type

**DOI:** 10.1002/alz70861_108110

**Published:** 2025-12-23

**Authors:** Sophie Roth, Laura Schlieker, Sayuri Hortsch, Joana Amorim Freire, David Caley

**Affiliations:** ^1^ Roche Diagnostics International AG, Rotkreuz, Zug Switzerland; ^2^ Roche Diagnostics GmBH, Penzberg, Bavaria Germany; ^3^ Staburo GmBH, Munich, Bavaria Germany; ^4^ Roche Diagnostics GmbH, Penzberg, Bavaria Germany; ^5^ Roche Diagnostics International, AG, Rotkreuz, Zug Switzerland; ^6^ Roche Diagnostics Ltd, Burgess Hill, West Sussex UK

## Abstract

**Background:**

This exploratory study investigates differences among patients enrolled in a clinical study based on referral type (self‐referred, doctor‐referred, or other), considering demographics, family history of Alzheimer's disease (AD) and other causes of symptoms, life circumstances, and clinical care factors.

**Method:**

Patients (*n* =608) were classified into three referral groups: self‐referred, doctor‐referred, and other. Data on demographics, family history of AD and other causes of symptoms, life circumstances, and clinical care factors were available. Descriptive analysis compared baseline characteristics across groups using Fisher’s exact test to derive *p* ‐values. Multivariable logistic regression analysis identified factors associated with referral type (self‐ and doctor‐referred).

**Result:**

Results indicate notable variations in patient characteristics and clinical factors between referral groups. In the group comparisons, there were no significant differences in age, gender, race, and number of previous visits between doctor‐referred and self‐referred patients. Self‐referred patients generally had higher socioeconomic status compared to doctor‐referred patients. Significant differences were observed in referral duration, with shorter referral durations for doctor‐referred patients compared to self‐referred patients (*p* <0.0005). Additionally, the type of doctors previously seen by the patient significantly differed between the two groups (*p* <0.0005). A higher number of self‐referred patients presented in primary care first than directly to specialists. 59% of patients (*n* =359) indicated a family history of AD and/or dementia. Logistic regression showed that this did not have a significant impact on the referral type. The three factors that did have a significant impact on the referral type were socioeconomic status, level of education and type of doctor seen previously (*p* <0.05).

**Conclusion:**

Understanding differences in patient characteristics based on referral types can provide insights into removing barriers to diagnosis and improving equitable diagnostic access. Current results explore the role of the patient's social environment, such as level of education, and how it can influence actionability on self‐presentation to medical care. Furthermore, doctor referred patients seem to have more access to the healthcare system, with shorter times between doctor visits. Future studies should focus on potential solutions for implementation of measures that improve access to timely and accurate diagnosis.

